# Supplementary material for: The molecular components of the anti-inflammatory cholinergic pathway are extrasplenic
Source: PLoS One. 2025 Sep 5;20(9):e0331707. doi: 10.1371/journal.pone.0331707 (PMC12412977; doi:10.1371/journal.pone.0331707)
Supplement: S1 Fig — (DOCX) [file pone.0331707.s001.docx]

**Supplementary Figure 1.**

Table of qPCR data from five wild-type male mice treated with a high dose of LPS (15 mg/kg, i.p.). Mice were sacrificed 1 hour and 30 minutes post-injection. Spleens were collected and processed for qPCR analysis as described in the main manuscript. Expression of *Chat* and *Adrb2* (used as a positive control gene) was assessed. Chat was undetectable in all samples.

|  |  |  | **Ct** | **Ct** |
| --- | --- | --- | --- | --- |
| **ID** | **Sample** | **18S** | **chat** | **adrb2** |
| L 1 | spleen | 10.14774 | 0 | 24.50188 |
| L 2 | spleen | 9.553129 | 0 | 24.46542 |
| L 3 | spleen | 10.74483 | 0 | 23.78975 |
| L 4 | spleen | 10.63851 | 0 | 24.16176 |
| L 5 | spleen | 10.52321 | 0 | 22.22133 |
