# Supplementary material for: The molecular components of the anti-inflammatory cholinergic pathway are extrasplenic
Source: PLoS One. 2025 Sep 5;20(9):e0331707. doi: 10.1371/journal.pone.0331707 (PMC12412977; doi:10.1371/journal.pone.0331707)
Supplement: Attachment — Submitted filename: rebuttal.docx [file pone.0331707.s003.docx]

Additional Editor Comments:

The manuscript has been reviewed by two referees, both of which request to revise it in terms of each comments and critisism. They specifically address the request to re-evaluate the accuracy regarding the conclusion. Since the spleen does neither express Chat mRNA nor alpha7 nAChR at all in residential immune cells, and therefore, it should be logical that ACh does not come from the spleen, rather from the vagus nerve. However, if so, vagus nerve stimulation may elevate local AChs in several tissues other than the spleen; moreover, even the splenectomized mice subjected to LPS load could be rescued by the stimulation as well as non-splenectomized mice. Based on referees' and editor's comments they need to conduct another experiment in order to consolidate their speculation and conclusion.

Thank you for the helpful and constructive feedback. We have revised our submission to address the concerns raised by both the editors and reviewers. Based on our data, we do not believe that acetylcholine (ACh) originates from either the spleen or the vagus nerve, as we did not observe any ChAT-positive innervation in our analyses (see Fig. 6B; see also PMID: 34729780). Therefore, we do not believe that experiments involving splenectomized models would be informative. That said, we have included new experiments as requested by Reviewer #2. We hope the revised manuscript is found to be significantly improved.

Please note that we amended our manuscript to satisfy Plos One recommendation. Changes are as follows:

Acknowledgment: We are grateful to Jenny Lee for her assistance with RNAscope studies in the early phase of this project. This basic research study was not pre-registered.

Financial disclosure: LG: NIH P01DK119130 (CNS mechanisms linking exercise training with energy balance and metabolism, Core C). The funders had no role in study design, data collection and analysis, decision to publish, or preparation of the manuscript.

Reviewers' comments:

Reviewer's Responses to Questions

Comments to the Author

1. Is the manuscript technically sound, and do the data support the conclusions?

The manuscript must describe a technically sound piece of scientific research with data that supports the conclusions. Experiments must have been conducted rigorously, with appropriate controls, replication, and sample sizes. The conclusions must be drawn appropriately based on the data presented.

Reviewer #1: Yes

Reviewer #2: Partly

________________________________________

2. Has the statistical analysis been performed appropriately and rigorously?

Reviewer #1: Yes

Reviewer #2: Yes

________________________________________

3. Have the authors made all data underlying the findings in their manuscript fully available?

The PLOS Data policy requires authors to make all data underlying the findings described in their manuscript fully available without restriction, with rare exception (please refer to the Data Availability Statement in the manuscript PDF file). The data should be provided as part of the manuscript or its supporting information, or deposited to a public repository. For example, in addition to summary statistics, the data points behind means, medians and variance measures should be available. If there are restrictions on publicly sharing data—e.g. participant privacy or use of data from a third party—those must be specified.

Reviewer #1: Yes

Reviewer #2: Yes

________________________________________

4. Is the manuscript presented in an intelligible fashion and written in standard English?

PLOS ONE does not copyedit accepted manuscripts, so the language in submitted articles must be clear, correct, and unambiguous. Any typographical or grammatical errors should be corrected at revision, so please note any specific errors here.

Reviewer #1: Yes

Reviewer #2: Yes

________________________________________

5. Review Comments to the Author

Please use the space provided to explain your answers to the questions above. You may also include additional comments for the author, including concerns about dual publication, research ethics, or publication ethics. (Please upload your review as an attachment if it exceeds 20,000 characters)

**Reviewer #1:** The manuscript is well written. Data are clear. This reviewer has a few comments on this manuscript.

The authors proposed the alternative model of the anti-inflammatory cholinergic pathway (shown in Figure 9) based on their mRNA expression data alone. Since no functional investigations were performed, the discussion section appears a bit speculative.

Thank you for the helpful and constructive feedback. We agree that our discussion was too speculative and wordy. Therefore, we revised it to remove entirely the speculative parts not directly linked to our own data.

The conclusions are based solely on mRNA expression data. Since detectable mRNA levels do not always correlate with protein abundance, it would be interesting to assess protein expression to strengthen the authors’ proposal.

We appreciate the suggestion to assess the α7 nicotinic acetylcholine receptor (α7 nAChR) protein. While this would indeed be of interest, to our knowledge, there are currently no specific and well-characterized antibodies available for this receptor. Unfortunately, the reliability of most commercially available antibodies targeting G protein-coupled receptors (GPCRs), including α7, remains a significant concern. This issue has been extensively documented by us and others in the field (see PMID: 37264690; 31080409). ChAT protein has been examined in the past by many investigators in the past and we do not wish to repeat this information. While there are reliable antibodies against ChAT, immunodetection of ChAT remains challenging, which is also an issue we discussed in detail in the past (PMID: 23749724; 34729780). In other words, we feel strongly that examining proteins is simply not feasible now.

**Reviewer #2:** The author concerned anti-inflammatory cholinergic pathway interacted between cholinergic vagal nerves and splenic immune cells so they proposed a new model of the cholinergic anti-

inflammatory pathway that highlights the roles of extrasplenic cholinergic signaling. This is a very interesting paper but there are major issues as shown below.

Thank you for the helpful and constructive feedback. We addressed your concerns to the further extent possible as explained below. In particular, we extensively revised our discussion which both reviewers found too speculative.

Major points

The manuscript needs to be reorganized and rewritten to ensure logical and appropriate placement of all content. Some information is presented in inappropriate sections while missing from where it would be most relevant. We apologize for the lack of clarity. Please find a revised manuscript which hopefully addresses your concerns (see below for details).

The descriptions of statistical analysis are very poor. The information of statistical analysis is need to provide evidence of statistical significance (determination of p-value, F-value, main effect, interaction, etc.) in the methods and the result sections not in the figure legend. We agree to reorganize our results according to reviewer #2’s comments. Results with statistics were moved from legends to the main manuscript. We are grateful to Reviewer #2 for pointing out the issues.

The manuscript lacks logical coherence in the conclusion and the experimental results are not discussed in sufficient depth to support the conclusions. For example, what mechanism about LPS-induced decreasing of the Adrb2 mRNA expression in the brainstem and spleen? The interpretation of the results is needed in the discussion section. There is insufficient interpretation or inference based on the presented data in the discussion section. We agree and our revised manuscript discusses potential mechanisms through which LPS regulated Adrb2 based on the available literature (see PMID: 19167076; see also our revised discussion).

It might be helpful to rewrite the section by linking each experimental result with previous reports to build a logical hypothesis, and then summarize the overall findings using Figures 8 and 9 in the discussion section. Our manuscript was revised to be clearer, less speculative, and more logical.

Only findings from present study should be described in the conclusions and references may be avoided in the conclusion section. We extensively revised our discussion to focus on our own findings as suggested by Reviewer #2.

The description in the figure 8 lacks of specific explanation each figure (A-E). Readers need more details of the figure not URL. We agree and we revised figure 8 accordingly.

Some of the description in the figure 9 is not the explanation of the figure. The author should reorganize the figure legend. We agree and we revised figure 9 accordingly.

LPS-induced decreasing gene expression of Adrb2 in the brainstem and spleen were well established by qPCR analysis in wild type mice. Several experiments are needed to explain that Chrna7 and Chat mRNA expression is not influenced by LPS injection (several dose of LPS and long-time course). Given that the anti-inflammatory effects of vagus nerve stimulation (VNS) manifest rapidly—typically within minutes to a few hours—we did not deem it necessary to assess the time course of Chrna7 expression over an extended period. The time point selected in our study (1.5 hours post-LPS) aligns with that used in the seminal work describing the cholinergic anti-inflammatory reflex (2 hours post-LPS; PMID: 12508119). Furthermore, it is important to note that multiple studies have demonstrated that VNS can suppress inflammation when applied prior to LPS administration (see discussion in PMID: 30396599). This strongly suggests that Chrna7 and Chat must already be expressed prior to immune challenge. Cells expressing α7 nicotinic receptors and capable of releasing acetylcholine are therefore expected to be present at baseline, before LPS administration.

Importantly, the anti-inflammatory cholinergic pathway was originally described using a much lower dose of LPS (0.1 mg/kg, i.p.) by Wang et al. (PMID: 12508119). Therefore, it is unlikely that the moderate LPS dose (1 mg/kg, i.p.) used in our experiments was insufficient to induce Chat expression in the spleen. However, we agree that it is scientifically relevant to explore whether a very high dose of LPS could influence Chat expression. To address this point, we conducted additional qPCR control experiments using a very high LPS dose (see new **supplementary data**). Notably, Chat remained undetectable regardless of the dosage.

Fluorescent RNAScope assays revealed that the expression of Chrna7 using is restricted to neurons. The expression of Chrna7 in the afferent neurons is very interesting, so the description of Chrna7 mRNA expression in the vagal and spinal afferent neuron and the involvement of these neurons in the anti-inflammatory pathway is need in the discussion section. We agree with the reviewer. We hope that our revised version discusses in enough detail the issue of neuronal Chrna7.

In the result section about the figure 5, the description “the expression of Chrna7 mRNA in the spleen was not significantly different between genotypes, we concluded low levels of Chrna7 in the spleen are attribute to a slight sample contamination during dissection an/or tissue processing.” is inappropriate. What means sample contamination? The phrases impress reduced reliability of the experimental data. I recommend verifying reproducibility with large sample size of mice. The expression of Chrna7 is not detected by using RNAScope assay, so the reason about this discrepancy is needed in the discussion. We apologize for our poor choice of words (e.g. contamination). We rewrote our text to avoid further confusion. We did not mean to say that our samples were technically “contaminated”, but that residual Chrna7 detection by qPCR may be due to a very small number of cells that are not undetectable by histology.

Minor points

How about ChAT-positive cell body in the nodose ganglion? In the figure 6, the authors did not mention the ChAT-positive neuronal cell body may be exist in the nodose ganglion. To ensure the validity of the study, it should be mentioned in the result section. This is a good question. As noted in our Chat-GFP data, we did not see any Chat-positive cell bodies in the nodose ganglion, but only axons. This is now better explained in our revision.

According to submission guideline, in the text, the reference number cite in square brackets (e.g., “We used the techniques developed by our colleagues [19] to analyze the data”). You must change the description of the reference number. We modified the formatting of our references.

The words “vagal afferents” is not suitable, so change to “vagal afferent neurons” in the line 199. We agree and modified our text.

In the legend of Figure 6, what is mean of the description “saline and LPS”? We apologize for the mistake and modified the manuscript.

Some description in the figure legend contains the appropriate content for the methods, so move and rewrite in the appropriate section. We modified our text.

The word “mRNA” is mentioned in the description about data from qPCR and RNA scope. We agree and modified our text.

Change “Chrn7” to “Chrna7” in the page18, line379, 381, 383, 386, 387, 386. Thank you for catching these errors. We corrected our text.

Change “microscopy” to “microscopic analysis” in the page18 line392. We agree and modified our text.

________________________________________

6. PLOS authors have the option to publish the peer review history of their article (what does this mean?). If published, this will include your full peer review and any attached files.

If you choose “no”, your identity will remain anonymous but your review may still be made public.

Do you want your identity to be public for this peer review? For information about this choice, including consent withdrawal, please see our Privacy Policy.

Reviewer #1: No

Reviewer #2: No

________________________________________
