# Supplementary material for: The molecular components of the anti-inflammatory cholinergic pathway are extrasplenic
Source: PLoS One. 2025 Sep 5;20(9):e0331707. doi: 10.1371/journal.pone.0331707 (PMC12412977; doi:10.1371/journal.pone.0331707)
Supplement: Attachment — Submitted filename: Response to Reviewers.docx [file pone.0331707.s004.docx]

**Response letter**

“The authors have almost revised original version according to the referees suggestion. however,

they have left several points unrevised despite the Referee 2 concerns., especially, 1) p values in figure legends depicted by asterisk not yet added, 2) references in the conclusion sentence, and 3) several typos in methods (Line 86, 131, and 246 in the revised version). Therefore, they need to revise them.”

“Additional Editor Comments:
The authors extensively revised the original manuscript based on the referees comments, and the speculative parts are fully revised. However, several parts which the referee pointed out are left not revised. Moreover, several typos have been still observed. For example, 1) Line 86, "since all our procedures were terminal. are listed". Does this mean that "since our procedures were terminal and are listed"?; 2) Line 131, "Prevalidated taqman assays were Mm01317884_ml (Chrna7)" Does this mean that "Prevalidated Taqman assays were performed using primers of Mm01317884_ml (Chrna7)"?; 3) Line 246, "(72)" should be altered to [72]. In addition, the authors need to re-revise the manuscript under the consideration of the referee 2 suggestions including statistical explanation in figure legends (p values) and deletion of references in a conclusion sentences.”

Dear editors,

We addressed previously raised concerns as explained below. Changes are marked-up in our revised manuscript. All typos were corrected. In addition, P values corresponding to the asterisks in each graph were added to the legends.

“Review Comments to the Author

**Reviewer #1:** Although this reviewer still has an interest in the protein expression, this reviewer understood the author's concerns regarding antibodies.

This reviewer considers the current version of the manuscript to be acceptable for publication.

We thank Reviewer 1 for his/her helpful comments throughout the revision process.

**Reviewer #2:** Comments to the Author

The authors have addressed majority of the concerns and the manuscript is substantially improved. I especially appreciate the manuscript was revised according to our concerns.

We thank Reviewer 2 for his/her helpful comments throughout the revision process.

There are several concerns remaining:

Major points

As I mentioned in my previous comment, it seems that it has not been fully understood. The following two points are presented below.

1. The author must read “PLOS ONE Statistical Reporting Guidelines“ carefully and revise the statistical analysis in the material and method, results and figure legend. Information about which statistical methods were used for which data, and which parameters were used to assess the effects with ANOVA, should be described in the methods section. Although statistical results are revised and presented in the results section, the definition of asterisks should be included in the figure legend.

As requested by reviewer 2, we added a section describing our statistical reporting.

2. Descriptions of inferences and interpretations are not appropriate for the results section. Please move these statements found in the results section to the discussion section. The results section should contain only the results and no other content.

We deleted interpretations and comments from the results sections whenever appropriate.

Minor points

As I mentioned before, citations are not necessary in the Conclusion section.”

We apologize for the misunderstanding. We have removed the citations included in the conclusion sentence.
